# Survival after cardiac arrest secondary to high-risk pulmonary embolism without reperfusion therapies: A case report

**DOI:** 10.1097/MD.0000000000016651

**Published:** 2019-08-02

**Authors:** Cai-Yun Xu, Jia-Fu Song, Li-Hong Yao, Hui-Ling Xu, Ke-Xi Liu

**Affiliations:** a Department of Intensive Care Medicine; b Department of Respiratory Medicine, The First People's Hospital of Lianyungang City, Lianyungang, Jiangsu; c State Key Laboratory of Respiratory Disease, The First Affiliated Hospital of Guangzhou Medical University, Guangzhou, China.

**Keywords:** cardiopulmonary resuscitation, case report, heart arrest, pulmonary embolism, withholding treatment

## Abstract

**Introduction:**

High-risk pulmonary embolism (PE) needs reperfusion therapies. However, it is difficult to make medical decisions when thrombolysis is contraindicated, though pulmonary embolectomy and percutaneous catheter-directed treatment (CTD) are recommended for these patients.

**Patient concerns:**

We reported here a case of high-risk PE patient with cardiac arrest (CA), vertebral compression fracture, as well as scalp and frontal hematoma.

**Diagnosis:**

The diagnosis of PE was based on computed tomography pulmonary angiography (CTPA) which demonstrated filling defects in the right and left pulmonary arteries.

**Interventions:**

Cardiopulmonary resuscitation was performed until the patient returned to idioventricular rhythm 3 minutes after admitted. She suffered another half-hour of hemodynamic disturbance after her shock improved 3 days later. The diagnosis of PE was confirmed by CTPA at that time. The patient did not receive any reperfusion therapies because hemoglobin decreased significantly. Moreover, anticoagulation was postponed for 2 weeks when bleeding appeared to be stopped. She received overlapping treatment with low molecular weight heparin and warfarin for 5 days then warfarin alone and discharged.

**Outcomes:**

She was discharged with normal vital signs and neurologically intact. She received anticoagulant therapy with warfarin and international normalized ratio regularly monitored after she was discharged, moreover, the pulmonary artery pressure turned normal, as determined by transthoracic echocardiography 1 month later. The warfarin treatment was discontinued after 12 months and no evidence of recurrence was seen until recently.

**Conclusions:**

This is the first case report of PE combined with CA that did not receive reperfusion therapy. We hypothesized that there was a spontaneous resolution in pulmonary emboli.

## 1 Introduction

Patients with pulmonary embolism (PE) presenting with shock or hypotension are at high risk of in-hospital death. In some cases, the first presentation of PE may be sudden death. Besides hemodynamic and respiratory support, thrombolysis and anticoagulation therapy should be immediately given to these patients as the preferred mode of initial anticoagulation.^[1]^ PE with vertebral body compression fracture is a contraindication to thrombolysis and anticoagulation for drug-related subdural hemorrhage may occur subsequently. However, according to the 2014 European Society of Cardiology and the guidelines in China, most contraindications to thrombolysis and anticoagulation should be considered relative in patients with high-risk PE. Do we need to apply thrombolytic and anticoagulant therapy for this patient with vertebral compression fracture under such guidance? The answer may be controversial. Some case reports indicate that anticoagulation alone could cause severe subdural hemorrhage and may even lead to paraplegia in patients with compression fracture.^[2–5]^

Surgical pulmonary embolectomy and percutaneous catheter-directed treatment (CTD) are recommended for patients in whom thrombolysis is contraindicated.^[1]^ Implantation of a temporary vena cava filter (VCF) is also an alternative method to overcome the period of absolute contraindications for anticoagulation. Yet these operations have their complications and risks, and may costs higher, which sometimes patients and their family cannot understand, so it is very difficult to make clinical decisions. Here we presented an acute PE case combined with cardiac arrest (CA) and compression fracture of thoracic vertebrae as a result of a fall followed by loss of consciousness. She survived without any reperfusion therapies.

## 2 Case report

**Figure 1:**
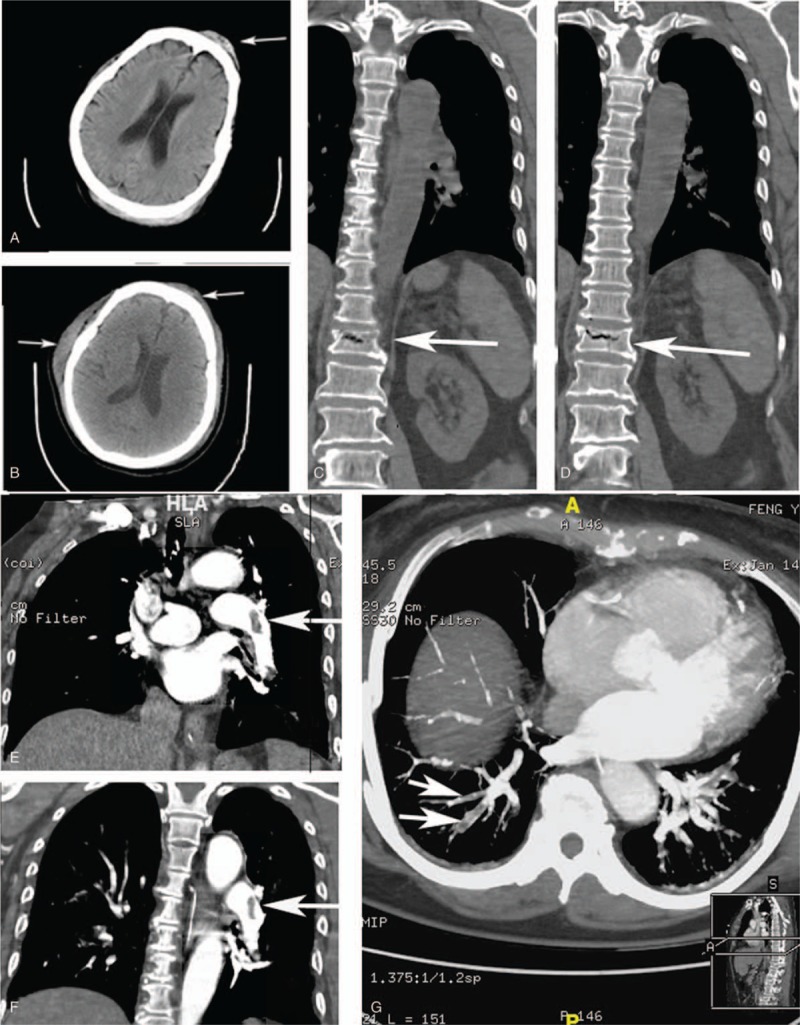
CT image changes in clinical course. (A and B) Head CT scan reveals scalp and frontal hematoma in the first and the third day. (C and D) Thoracic spine CT indicates T12 compression fracture. (E and G) CTPA shows PE with multiple filling defects in the right and left pulmonary arteries. CT = computed tomography, CTPA = computed tomography pulmonary angiography, PE = pulmonary embolism.

The patient was a 74-year-old woman; she was admitted to the emergency department because of chest tightness and loss of consciousness for 30 minutes. She felt chest tightness the moment she got up. She lost her balance and fell down from the 1-m-high stair, hitting her forehead. Her family found her unconsciously immediately. One minute later her consciousness recovered and she complained about chest distress. She was found CA after the ambulance took her to the emergency department. Cardiopulmonary resuscitation was performed; she was intubated and mechanically ventilated. She returned to idioventricular rhythm (cardiac junctional escape rhythm compared with complete right bundle block) after 3 minutes, but was still in deep coma, with no spontaneously breathing. Blood gas analysis revealed metabolic acidosis (pH: 7.18, pCO_2_: 42 mm Hg, pO_2_: 136 mm Hg, HCO3^−^: 15 mmol/L, Lac >15 mmol/L). D-dimer level was 86,451 ng/mL; troponin T level was 1.03 ng/mL (reference range <0.01 ng/mL). Transthoracic echocardiography (TTE) indicated normal cardiac structure and function except that pulmonary artery pressure was 45 mm Hg (reference range <30 mm Hg). Head, chest, and abdomen computed tomography (CT) scan revealed scalp and frontal hematoma and T12 compression fracture (Fig. 1). The diagnosis was CA, although the etiology was unknown. Mild hypothermia and cerebral dehydration therapies were also given. Her consciousness returned after 8 hours. She was therefore sent to intensive care unit (ICU) after extubation. She had a 2-year history of type 2 diabetes with the fasting blood glucose levels ranging between 8 and 10 mmol/L, but did not take any medications. She had got a cold during those days with cough and dyspnea but no special medical record. Her mood was sad and had a bed rest for 3 days.

She was anuric upon admission to ICU with serum creatinine rising up to 180 mmol/L. The vital signs were normal, and even oxygen therapy was not needed. In order to avoid further worsening of renal function, computed tomography pulmonary angiography (CTPA) was delayed; the patient had clinically suspected PE although bilateral compression ultrasonography revealed no deep-vein thrombosis. A bandage was applied with pressure over the head to prevent exacerbation of scalp hematoma. Her hemoglobin concentration decreased from 11.5 g/dL to 6.7 g/dL 2 days after admission to ICU. In addition, the shock had been controlled (with urinary output increasing, creatinine and lactate levels returning to normal); the D-dimer level dropped to 5601 ng/mL. Unfortunately, her oxygen saturation suddenly decreased to 85% with blood pressure reducing from 140/70 mm Hg to 90/60 mm Hg for about half an hour and she fell unconscious again on day 3 after admission. For the above reasons, she was reintubated and ventilated. CTPA and head CT scan were performed. The diagnosis of PE was confirmed by CTPA which demonstrated filling defects in the right and left pulmonary arteries (Fig. 1), and no new bleeding sites were discovered by CT (Fig. 1). The D-dimer level increased again. She was given 4U red blood cell transfusion. We thought the risk of massive hemorrhage might still outweigh the benefits of anticoagulant and fibrinolytic therapies. However, are our decisions really right? On this account, multi-disciplinary team work was carried on. Experienced cardiologists thought we should not use systemic thrombolysis, and anticoagulant therapy should also be delayed. Pulmonologists deemed that anticoagulation should be given at once. The spine surgeon requested the patient to have a strict bed rest in order to prevent fracture fragments redisplacement. In the opinion of interventional radiologists and vascular surgeons, the patient needs VCF. However her family refused VCF due to financial shortage. Moreover, her family also let us ensure that threatening bleeding and any other complications will not be incurred because they had to pay about $150 a day for medical expenses. As a result, the patient did not receive any reperfusion therapies. Luckily, she regained her consciousness and passed the spontaneous breathing trial the fourth day after reintubation only with the help of supportive therapy. Moreover, anticoagulation was postponed till 2 weeks later when active bleeding might appear to stop. The patient remained in stable conditions and was transferred out of ICU 2 weeks after admission. She received an overlapping therapy with low molecular weight heparin and warfarin for 5 days then warfarin alone and discharged. International normalized ratio was regularly monitored after she was discharged from hospital. Her family refused CTPA rescreening, but TTE was performed after discharge. The pulmonary artery pressure returned to normal 1 month later and stabilized during the 15-month follow-up period. The warfarin treatment was discontinued 12 months after hospital discharge and no evidence of recurrence was seen until recently.

## 3 Discussion

There are few data derived from clinical trials that could guide therapeutic decisions for CA secondary to PE. We conducted a detailed search of the literature in English published before October 2018 in PubMed using the search criteria “pulmonary embolism” and “cardiac arrest” and also manually searched the cases in relevant references. We finally found 44 articles about therapy, of which 42 are case reports, 1 is small sample randomized controlled trail research, and the other is a retrospective study. Of these, 29 were treated with thrombolysis; the other 15 were treated with surgical pulmonary embolectomy or CTD. In a word, all patients in these studies were treated with revascularization therapy. However, the patient in this case was not. Why did we make such a decision? On the one hand, the patient had thoracic vertebral compression fractures and active bleeding, which are contraindications to anticoagulation, not to mention thrombolysis. On the other hand, though surgical pulmonary embolectomy and CTD are recommended for patients in whom thrombolysis is contraindicated, both methods are rarely performed in patients with PE in most hospitals of China, including our hospital.

Of course, we can implant a VCF into our patient to overcome the period of absolute contraindications for anticoagulation. We conducted a search of the literature in PubMed using the search criteria “pulmonary embolism” and “contraindication of anticoagulation” and finally found that VCF is a potential strategy to prevent recurrence of PE in patients with contraindications for anticoagulant therapy^[6,7]^ and most patients with PE like our case choose to implant a VCF. Conversely, withholding treatment may be associated with fewer adverse events than treatment with anticoagulants or a VCF in patients with nonmassive acute PE.^[8]^ It is a possible strategy for the management of patients with a high risk of bleeding or other contraindications to anticoagulants. Initially, we recommended implanting a VCF for the patient, but her family refused the treatment due to financial shortage and potential risks.

Fortunately, the patient managed to survive. We believe that her recovery was due to spontaneous resolution of pulmonary emboli. We think so for the following reasons. Firstly, D-dimer is a fibrin degradation product formed during the lysis of a thrombus. Researches suggest that the D-dimer levels decreased rapidly within days in the PE patients under appropriate therapy.^[9–11]^ Although our patient did not receive reperfusion therapy, the D-dimer level did decrease rapidly, indicating activation of endogenous fibrinolytic system. Secondly, patients with obstructive shock secondary to massive PE are usually difficult to treat unless the obstruction was relieved,^[12]^ but she regained spontaneous circulation 3 minutes after CA, which implied spontaneous resolution of pulmonary emboli. Thirdly, the occurrence of CA suggested a high probability of major pulmonary artery blockage, but the area of pulmonary artery filling defect on CTPA of our patient did not seem to cause shock or sudden death, which may also implied spontaneous resolution of pulmonary emboli before CTPA had performed.

As far as we know, this is the first case report of PE combined with CA that did not receive reperfusion therapy. We hypothesized that there was a spontaneous resolution in pulmonary emboli. Because the arrest time is short, but the D-dimer level decreased rapidly and CTPA scanning did not indicate massive PE.

## Acknowledgment

The authors thank all nurses and staff of our department for their precious help and cooperation.

## Author contributions

**Conceptualization:** Cai-Yun Xu, Jia-Fu Song.

**Data curation:** Cai-Yun Xu, Jia-Fu Song, Hui-Ling Xu, Ke-Xi Liu.

**Investigation:** Cai-Yun Xu, Jia-Fu Song.

**Project administration:** Cai-Yun Xu, Ke-Xi Liu.

**Writing – original draft:** Cai-Yun Xu.

**Writing – review & editing:** Jia-Fu Song, Li-Hong Yao.
